# Predictive value of tumor microenvironment on pathologic response to neoadjuvant chemotherapy in patients with undifferentiated pleomorphic sarcomas

**DOI:** 10.1186/s13045-024-01614-w

**Published:** 2024-10-23

**Authors:** Jean Philippe Guegan, Nathan El Ghazzi, Julien Vibert, Christophe Rey, Lucile Vanhersecke, Jean Michel Coindre, Maud Toulmonde, Mariella Spalato Ceruso, Florent Peyraud, Alban Bessede, Antoine Italiano

**Affiliations:** 1 Explicyte, Bordeaux, France; 2 Department of Medicine, Early Phase Trials and Sarcoma Units, Institut Bergonié, 229 Cours de l’Argonne, Bordeaux, France; 3 Unversity Hospital Center, Clermont Ferrand, France; 4 DITEP, Gustave Roussy, Villejuif, France; 5 Department of Pathology, Institut Bergonié, Bordeaux, France; 6 Faculty of Medicine, University of Bordeaux, Bordeaux, France

**Keywords:** Soft tissue sarcoma

## Abstract

Undifferentiated pleomorphic sarcomas (UPS) represent a prevalent and aggressive subtype of soft tissue sarcomas (STS) in adults. Despite advancements in loco regional treatments, many patients with high grade STS, including UPS, develop metastatic disease. Neoadjuvant chemotherapy is a standard approach to mitigate this risk, but response variability necessitates refined patient selection strategies. This study investigated the correlation between UPS microenvironment and neoadjuvant chemotherapy response in resectable UPS. The NEOSARCOMICS study (NCT02789384) enrolled patients with resectable STS from six sarcoma centers in France. Patients received anthracycline based chemotherapy, followed by surgery. Histological response, gene expression profiling, and multiplex immunohistofluorescence were performed on baseline and post treatment tumor samples. Plasma proteomics was analyzed to identify biomarkers. Good responders to neoadjuvant chemotherapy showed enrichment in genes related to stemness and cell cycle regulation, while poor responders exhibited immune related gene enrichment. Proteomic profiling revealed immune pathway activation and downregulation of cell cycle pathways in non responders. Despite being associated with a good prognosis, high immune infiltration, particularly of CD8 + T cells and CD20 + B cells, predicts a poor response to neoadjuvant chemotherapy in UPS, suggesting the need for alternative therapeutic strategies for patients with inflamed UPS.Ongoing clinical trials are exploring the efficacy of combining chemotherapy with immune checkpoint inhibitors to improve outcomes.

**Supplementary Information:**

The online version contains supplementary material available at 10.1186/s13045-024-01614-w.

## To the Editor

Undifferentiated pleomorphic sarcomas (UPS) are a prevalent and aggressive subtype of soft tissue sarcomas (STS) in adults [1]. We have previously shown that there are two distinct UPS subgroups: immune high, with increased immune infiltrates and upregulated immune checkpoints, associated with lower metastatic relapse and better survival; and immune low, characterized by more gene copy number alterations, particularly in tumor suppressor genes, and a poorer prognosis [2].

Neoadjuvant chemotherapy is often used to reduce the risk of metastatic relapse in patients with high-grade UPS [3], though its variable efficacy necessitates better patient selection strategies. Studies in epithelial tumors have shown a link between the tumor microenvironment and chemotherapy response [4]. We hypothesize that UPS response to neoadjuvant chemotherapy is influenced by immune cell composition.

To confirm the prognostic value of the immune classification of UPS we previously identified [2], we first investigated whether the amount of tumor infiltrating immune cells influenced the risk of metastatic relapse and death in a cohort of 47 patients with UPS who underwent surgery for localized disease. Tissue microarrays of UPS samples were stained with the multiplex IHF panel combining CD8, CD14, CD20, CD45, CD68, cMAF and DAPI markers (Supplementary Methods, Supplementary Fig. 1A). The patients’ characteristics are described in Supplementary Table 1. We observed that patients with high SARCULATOR total score, e.g. low survival probabilities, were less infiltrated in immune cells and notably in CD8 + cells and M1 macrophages (CD68+/cMAF cells) (Supplementary Fig. 1B). Similarly, patients with high levels of CD20+, CD8+, CD14 + cells or M2 macrophages (CD68+/cMAF + cells) tend to have a better overall survival than UPS patients with lower infiltration (Supplementary Fig. 1C).

**Fig. 1:**
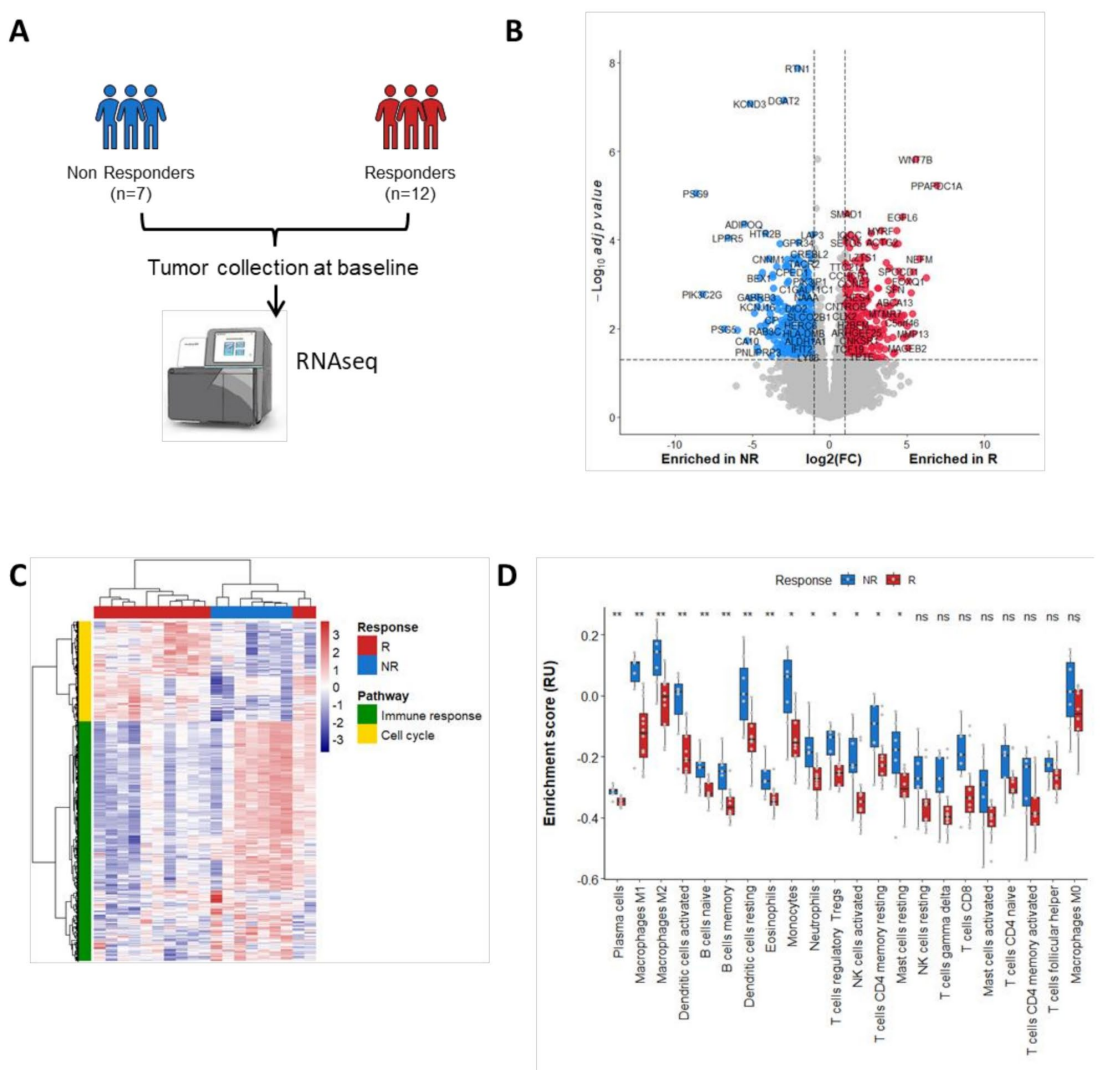
Response to neoadjuvant chemotherapy correlates with high proliferation and low immune infiltration phenotype. (**A**) Workflow of RNAseq experiment performed on baseline tumor samples from UPS patients treated with neoadjuvant chemotherapy. (**B**) Volcano plot representation of genes differentially expressed between responder (R) and non responder (NR) patients. (**C**) Heatmap visualization of Gene Ontology biological process enrichment scores. (**D**) Boxplot representation of immune cells estimation (CIBERSORT) according to response to neoadjuvant chemotherapy. P values were calculated using Wilcoxon tests

Then to decipher the impact of UPS microenvironment on response to neoadjuvant chemotherapy, we first performed gene expression profiling of baseline samples from 24 patients with resectable UPS who were treated with anthracycline based neoadjuvant chemotherapy and enrolled in the NEOSARCOMICS study (Supplementary Table 2, Supplementary Methods, Fig. 1A). Twelve patients had a good histological response after central blinded pathological assessment. The examination of differential gene expression between good responders and poor responders to neoadjuvant chemotherapy unveiled 1058 genes (Fig. 1B Supplementary Table 3).

The good responders group showed significant enrichment in genes related to stemness, cell cycle regulation, and oncogenesis (Fig. 1C, Supplementary Table 3). This included key genes like LHX8, involved in stem cell fate [5]; CCNE1, CDC25A, and CDK2, which regulate the G1/S cell cycle transition [6]; DNA polymerase genes (POLE, POLM, POLD1); and FGFR2, previously identified in the immune low UPS subgroup [2]. In contrast, poor responders exhibited enrichment in genes related to immune response pathways, such as type I IFN signaling and myeloid and lymphocyte activation, suggesting a strong immune presence in the tumor microenvironment (Fig. 1C, Supplementary Table 3). CIBERSORT analysis further revealed that poor responders were highly enriched in immune cells (Fig. 1D).

**Fig. 2:**
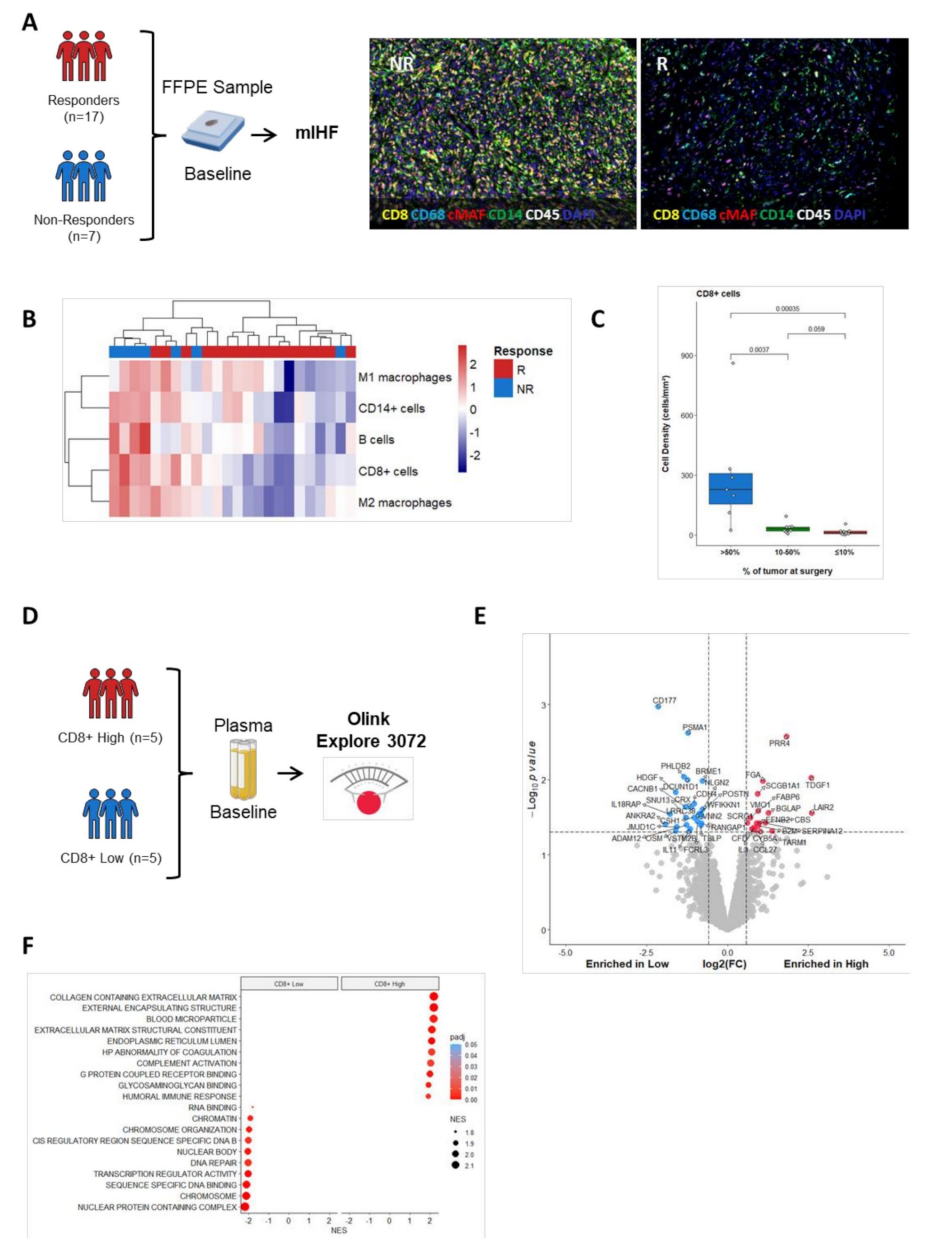
Poor response to neoadjuvant chemotherapy is associated with baseline immune infiltrates in UPS patients. (**A**) Workflow of multiplex IHF validation experiment (left). Representative images of tumor FFPE section of non responder (NR) and responder (R) patients stained with the multiplex panel CD8 / CD14 / CD20 / CD45 / CD68 / cMAF / DAPI. (**B**) Heatmap visualization of cell densities of indicated immune cell populations in UPS patients. (**C**) Boxplot representation of CD8 + cell densities according to percentage of tumors cell at surgery. P values were calculated using Wilcoxon tests. (**D**) Workflow of plasma proteomic analysis using Olink Explore 3072. (**E**) Volcano plot representation of protein differentially secreted at baseline between CD8 + high and low UPS patients. (**F**) Bubble plot of Gene Ontology terms enrichment

To visualize the difference in immune cell abundances between responders and non-responders and confirm gene expression data, baseline tumor samples were stained with the multiplex IHF panel CD8 / CD14 / CD20 / CD45 / CD68 / cMAF / DAPI (Supplementary Methods, Fig. 2A). Quantification of immune cell density confirmed that baseline samples from patients with a poor response to neoadjuvant chemotherapy tended to be highly infiltrated by immune cells (Fig. 2B and C). Analysis of plasma proteins (Supplementary Methods, Fig. 2D) differentially expressed at baseline between CD8 + High and Low UPS patients highlighted the upregulation of cell cycle pathways in patients with low immune infiltration (Fig. 2E F).

To assess the impact of neoadjuvant chemotherapy on the tumor microenvironment, we analyzed gene expression profiles from paired pretreatment biopsy and surgical specimens in responders (*n* = 3) and non-responders (*n* = 5) (Supplementary Fig. 2A). Differentially expressed genes at surgery varied significantly between the two groups (Supplementary Fig. 2B). Hallmark gene signature analysis showed that allograft rejection was elevated in responders, while TNFα and Wnt/βcatenin signaling were specific to non-responders (Supplementary Fig. 2C). Deconvolution analysis revealed increased infiltration of cytotoxic CD8 + T cells and CD20 + B cells in responders, but not in non-responders (Supplementary Fig. 2D). Additionally, plasma proteomics linked a good response to higher levels of CD5L and lower levels of GDF 15 (Supplementary Fig. 3).

While high immune infiltration of UPS correlates with better survival, it predicts poor pathological response to chemotherapy, emphasizing the complex role of the tumor microenvironment. A key factor may be the presence of M2 macrophages, enriched in non-responders, which are linked to chemoresistance by suppressing T cell function and promoting tumor survival [7, 8]. Regulatory T cells (Tregs) were also enriched in poor responders, mirroring findings in breast cancer, where Tregs are linked to poor chemotherapy responses [9, 10].

Although this study focused on immune infiltration, the stromal and extracellular matrix (ECM) components of the tumor microenvironment also likely affect chemotherapy response by acting as physical barriers to drug delivery [11]. Conversely, the immune low group may respond better to chemotherapy, with an enrichment of genes involved in cell cycle regulation and oncogenesis [2].

Our results could help stratify UPS patients by immune status for more personalized treatments. Our findings suggest that standard chemotherapy may not be optimal for immune high UPS patients. Combining chemotherapy with immune checkpoint inhibitors or targeting tumor-associated macrophages could offer better outcomes [12]. Ongoing studies, such as NCT04968106, are exploring chemoimmunotherapy combinations in high-grade UPS, with histological response as a primary endpoint, potentially advancing our understanding of immune-tumor interactions.

## Electronic supplementary material

Below is the link to the electronic supplementary material.

Supplementary Material 1

Supplementary Material 2

## Data Availability

No datasets were generated or analysed during the current study.

## Abbreviations

cMAF: Musculoaponeurotic Fibrosarcoma Oncogene Homolog
CD: Cluster of Differentiation
DAPI: 4’,6 Diamidino 2 Phenylindole
DMFS: Distant Metastases Free Survival
DNA: Deoxyribonucleic Acid
ECOG: Eastern Cooperative Oncology Group
ECM: Extracellular Matrix
EORTC STBSG: European Organisation for Research and Treatment of Cancer Soft Tissue and Bone Sarcoma Group
GDF 15: Growth Differentiation Factor 15
HRP: Horseradish Peroxidase
IHC: Immunohistochemistry
IFN: Interferon
mTOR: Mechanistic Target of Rapamycin
NCT: National Clinical Trial
NGS: Next Generation Sequencing
NPX: Normalized Protein Expression
OS: Overall Survival
PCR: Polymerase Chain Reaction
PEA: Proximity Extension Assay
PI3K: Phosphoinositide 3 Kinase
RNA: Ribonucleic Acid
SARCULATOR: Sarcoma Calculator
STS: Soft Tissue Sarcomas
TME: Tumor Microenvironment
TNFα: Tumor Necrosis Factor Alpha
Tregs: Regulatory T Cells
TSA: Tyramide Signal Amplification
UPS: Undifferentiated Pleomorphic Sarcomas
Wnt: Wingless related integration site
