# Supplementary material for: Predictive value of tumor microenvironment on pathologic response to neoadjuvant chemotherapy in patients with undifferentiated pleomorphic sarcomas
Source: J Hematol Oncol. 2024 Oct 23;17:100. doi: 10.1186/s13045-024-01614-w (PMC11515614; doi:10.1186/s13045-024-01614-w)
Supplement: Supplementary file 2 — Supplementary Material 2 [file 13045_2024_1614_MOESM2_ESM.docx]

***SUPPLEMENTARY METHODS***

***Trial design and oversight***

The NEOSARCOMICS study (NCT02789384) is a prospective biomarker study that has recruited patients with resectable soft tissue sarcomas (STS) from six specialized sarcoma centers in France. Participation in the study was contingent on a recommendation for neoadjuvant chemotherapy from a multidisciplinary tumor board. The clinical trial adhered to ethical guidelines, including the Declaration of Helsinki and Good Clinical Practices. This study was approved by the Central Institutional Review Board (Comité de Protection des Personnes Sud-Ouest III, Bordeaux, France) under protocol number ID-RCB n° 2015-A01969-40. All participants provided written informed consent.”Prior to enrollment, all patients provided written informed consent.

***Patients***

Main eligibility criteria included: Age ≥ 18 years; Eastern Cooperative Oncology Group (ECOG) performance status ≤ 1; Histological confirmation of soft-tissue sarcoma by central review; Full list of eligibility criteria is available in the protocol

***Study treatment***

All patients received treatment in accordance with the established standard of care. The chemotherapy regimen mandated inclusion of doxorubicin (dose range: 60 - 75 mg/m² on day 1) and ifosfamide (dose range: 2.5 - 3g/m² on days 1, 2, and 3), administered in 21-day cycles for up to 6 cycles preceding surgery. Following the completion of neoadjuvant chemotherapy, patients underwent surgical intervention, with the option of subsequent radiotherapy as deemed appropriate.

***Histopathology***

Diagnosis was made by an expert pathologist expert in the field of STS. To ensure accurate diagnosis of UPS, we employed a panel of immunohistochemical markers, including CD34, desmin, S100, and myogenin, to exclude other sarcoma subtypes and confirm the undifferentiated pleomorphic sarcoma diagnosis. All biopsies and resection specimens underwent assessment by an experienced sarcoma pathologist. Notably, the pathologists were blinded to radiological outcomes. For response evaluation, resection specimens were scrutinized in accordance with standard protocols. Parameters of interest included the total percentage of viable/stainable cells, with a corresponding final response score as per the EORTC-STBSG guidelines [12]. Additionally, the evaluation encompassed the total percentage of necrosis and the total percentage of fibrosis/hyalinization, hereafter referred to as 'fibrosis.' A good histological response was defined as the presence of less than 10% viable tumor in the resection specimen.

***RNA sequencingHaut du formulaire***

The transcriptomics analysis was performed on patients with available pre-treatment frozen samples as previously described [13]. Whole-RNA sequencing included aligning RNA sequences to the transcriptome and estimating gene expression. Gene expression counts were normalized using the Voom method to ensure statistical reliability [13]. RNA was extracted using the Rneasy Mini Kit (Qiagen), with NGS library preparation and sequencing performed by Integragen (France). Quality control involved Nanodrop and Qubit 2.0 assessments. Libraries were prepared using the NEBNext Ultra II mRNA-Seq Kit (NEB) and sequenced on the NovaSeq 6000 platform (Illumina), targeting 65 million paired-end sequences per patient.

Bioinformatics analysis utilized the Hg19/Gh37 human genome. Quality control and alignment were performed using FastqPairedEndValidator, Clumpify, Sickle, SeqPrep, Bowtie2, and Tophat2. PCR duplicates were removed using PicardTools. Gene expression counts were normalized using the Voom method, transforming count data into log2-counts per million (logCPM) and applying observational-level weights.

4o

***Multiplex-IHF assay***

Multiplexed immunohistofluorescence was performed using the following panel CD8/CD14/CD20/CD45/CD68/cMAF/DAPI as previously described. The following antibodies were used: CD8 (C8/144B, Dako), CD14 (EPR3653, Abcam), CD20 (L26, Ventana), CD45 (LCA, Cell Marque), CD68 (PG-M1, Dako) and cMAF (EPR16484, Abcam). Bound primary antibodies were detected using OmniMap anti-Rb HRP (760-4311, Ventana) and OmniMap anti-Ms HRP (760-4310, Ventana) detection kits followed by TSA opal fluorophores (Opal 480, Opal 520, Opal 570, Opal 620, Opal 690 and/or Opal 780, Akoya Bioscience). The slides were counterstained with spectral DAPI (Akoya Bioscience) and cover-slipped. The slides were scanned using the PhenoImager HT System (Akoya). Tumor areas were delineated in PhenoChart (Akoya Bioscience) by a pathologist and analyzed using inForm software (Akoya Bioscience, version 2.6.0) to segment the tissue and identify the cells. Cell segmentation was achieved using an object-based approach implemented in inForm software. DAPI staining specifically identified cell nuclei, while additional membrane marker staining further refined the segmentation of individual cells. The mean marker intensity was extracted for each cell and signal intensities were further normalized using the GaussNorm function from flowstat R package. Cells were finally phenotyped using a thresholding method in FlowJo (version 10.8.0).

***Plasma proteomics***

Plasma protein levels were quantified using the Olink Proximity Extension Assay (PEA) (Olink Proteomics AB, Uppsala, Sweden) according to the manufacturer's instructions. Briefly, matched pairs of antibodies conjugated to unique oligonucleotide sequences were used to target proteins of interest. In the presence of the target protein, the antibodies bind and their proximal oligonucleotides hybridize, generating a unique DNA reporter sequence. This reporter sequence was then amplified by polymerase chain reaction (PCR) and quantified using next-generation sequencing (NGS) on a Novaseq 6000 system (Illumina, Inc., San Diego, CA). The Olink® Explore 3072 panel was employed for protein profiling. Quality control and normalization of the data was performed using plate controls. Protein expression levels are reported as NPX values, a log2-scaled arbitrary unit where higher values indicate increased protein abundance. Further details on assay validation, including detection limits and precision data, can be found on the Olink website. Differential protein expression was performed using “Limma” R package (v3.60.3).

***Statistical analysis***

The primary objective of this study was to scrutinize the relationship between microenvironmental features in the pre-treatment biopsy and histological response. A secondary aim was to investigate the correlation between peripheral blood proteomics and histological response. Additional outcome parameters of interest encompassed distant metastases-free survival (DMFS) and overall survival (OS). DMFS was computed from the surgery date (disease-free status) to the occurrence of distant metastasis (DM), with patients diagnosed with synchronous DM excluded from this analysis. OS was calculated from the time of diagnosis to the last follow-up moment or the occurrence of death. SARCULATOR OS scores were calculated using SARCULATOR nomogram as previously described [14]

Survival rates were estimated through the Kaplan–Meier method. Patient categorization as "high" or "low" for different immune cell subsets was based on optimal cut-point values determined using the "survminer" R package (v0.4.9). Differences between groups were assessed using the Wilcoxon-Mann-Whitney test for continuous variables.

***Data Availability***

The datasets that support the findings of this study are not publicly available due to information that could compromise research participant consent. According to French/European regulations, any re-use of the data must be approved by the appropriate ethics committee. Individual participant data that underlie the results reported in this article can be shared upon request to the corresponding author (AI). Proposals may be submitted up to 36 months following article publication.

**SUPPLEMENTARY TABLES AND FIGURES**

**Supplementary Table 1. Patient characteristics (prognostic cohort n=47)**

|  | **n (%)** |
| --- | --- |
| **Median age** (years, range) | 63 (29 – 83) |
| **Gender** |  |
| Female | 19 (40.4%) |
| Male | 28 (59.6%) |
| **Tumor size** |  |
| ≤ 5 cm | 6 (12.8%) |
| > 5 cm and ≤10 cm | 23 (48.9%) |
| >10 cm | 18 (38.3%) |
| **Tumor grade** |  |
| G2 | 7 (14.9%) |
| G3 | 40 (85.1%) |
| **Tumor Depth** |  |
| Superficial | 5 (10.6%) |
| Deep | 42 (89.4%) |
| **Tumor site** |  |
| Lower limb | 32 (68.0%) |
| Upper limb | 6 (12.8%) |
| Trunk wall | 9 (19.2%) |

**Supplementary Table 2. Characteristics of the NEOSARCOMICS study population**

**(n = 24 )**

| **Characteristics** | **Patients** |
| --- | --- |
| **Age (years)** | |
| Median (range) | 64 (52-77) |
| **Sex** | |
| Women | 10 (42%) |
| Men | 14 (58%) |
| **WHO performance status** | |
| 0 | 18 (75%) |
| 1 | 6 (25%) |
| **Tumor location** | |
| Trunk wall | 3 (12.5%) |
| Upper limb | 5 (21%) |
| Lower limb | 16 (66.5%) |
| **Tumor depth** | |
| Deep | 22 (91.5%) |
| Deep and superficial | 2 (8.5%) |
| **Tumor size (mm)** | |
| ≤ 50 | 2 (8 %) |
| >50 and ≤100 | 12 (50%) |
| >100 | 10 (42%) |
| **Tumor Grade** | |
| 3 | 24 (100) |

**
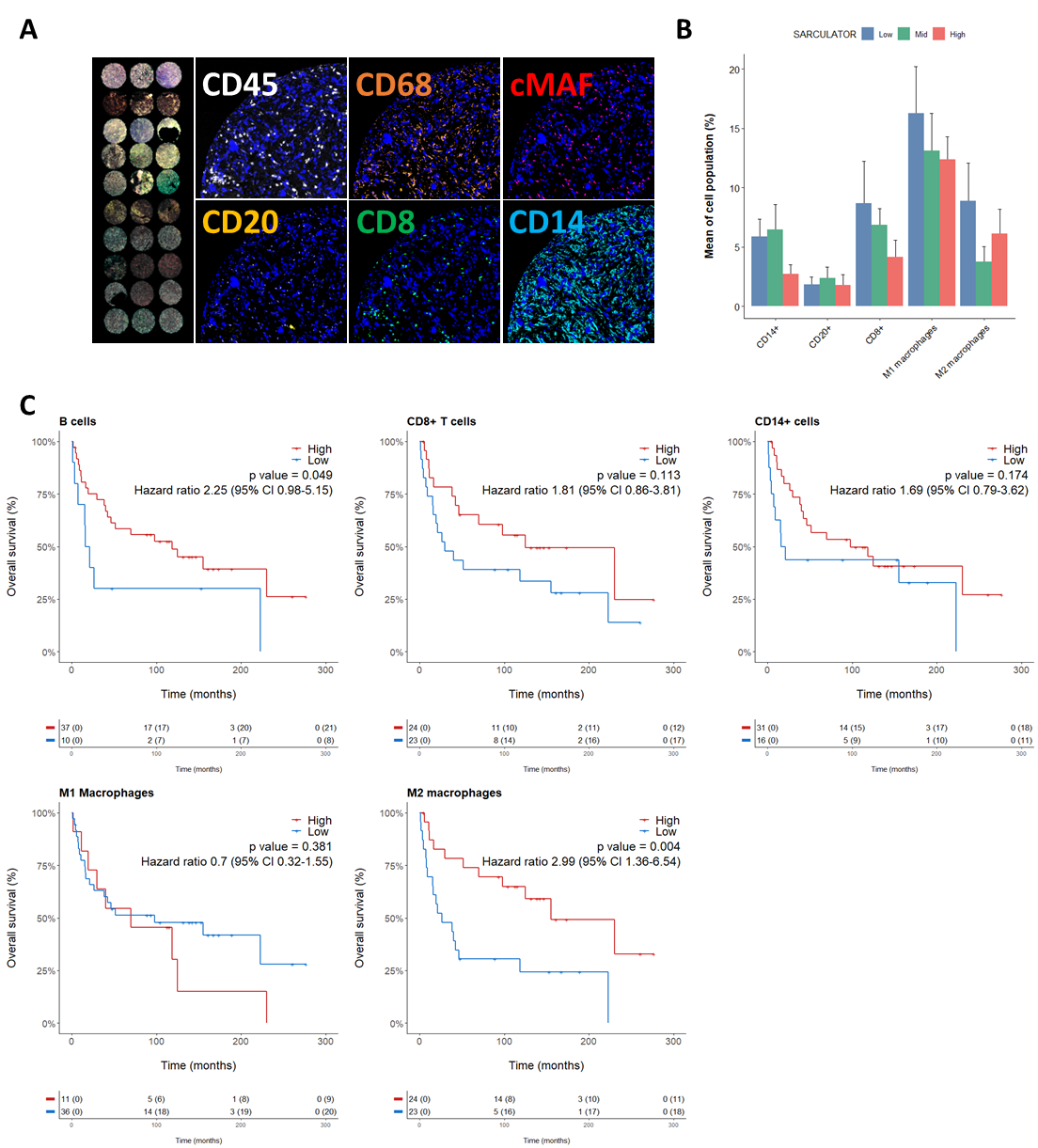
Supplementary Figure 1: Immune infiltration is associated with prognosis of UPS patients.** (A) Tissue microarrays of UPS tumor sections were stained with the multiplex IHF panel CD8/CD14/CD20/CD45/CD68/cMAF/DAPI. (B) histograms of mean cell density of identified immune cells according to SARCULATOR OS total scores. (C) Kaplan Meier curves of overall survival of patients classified as “High” or “Low” according to infiltration of indicated immune cell population. P values were calculated using log-rank tests.

**
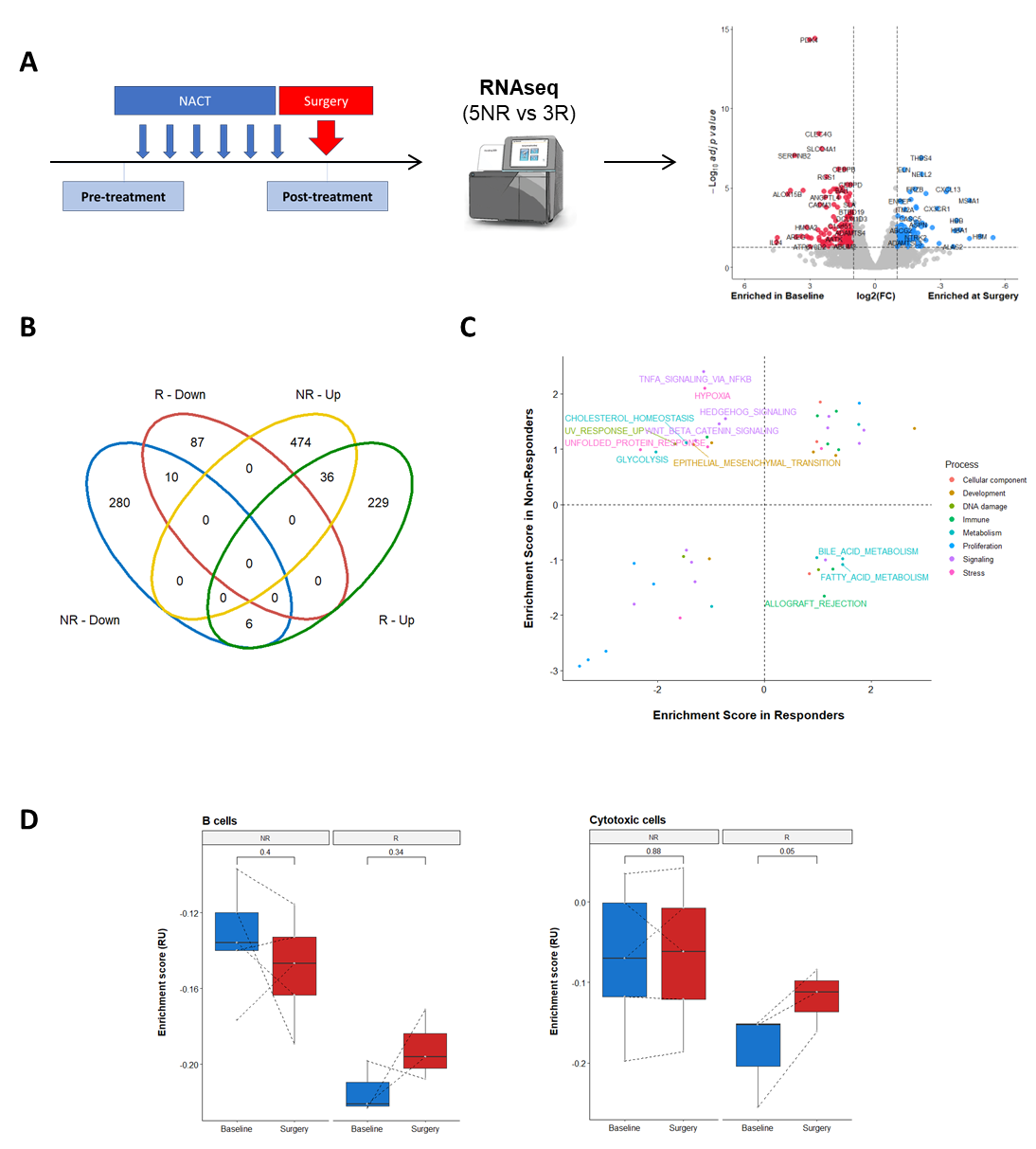
**

**Supplementary Figure 2: Response to neoadjuvant chemotherapy is associated with immune cell recruitment.** (A) Workflow of RNAseq experiment performed on pair samples collected at baseline and at surgery after neoadjuvant chemotherapy (NACT). (B) Venn diagram of genes significantly upregulated (Up) or downregulated (Down) in responder (R) and non-responder (NR) patients during neoadjuvant chemotherapy. (C) Visualization of normalized enrichment scores of Hallmark gene signature in responder and non-responder patients. Only significantly disregulated pathways are labeled. (D) Boxplot representation of “B cells” and “Cytotoxic cells” gene signature enrichment in responder and non-responder patients. P values were calculated using paired t-tests.

**
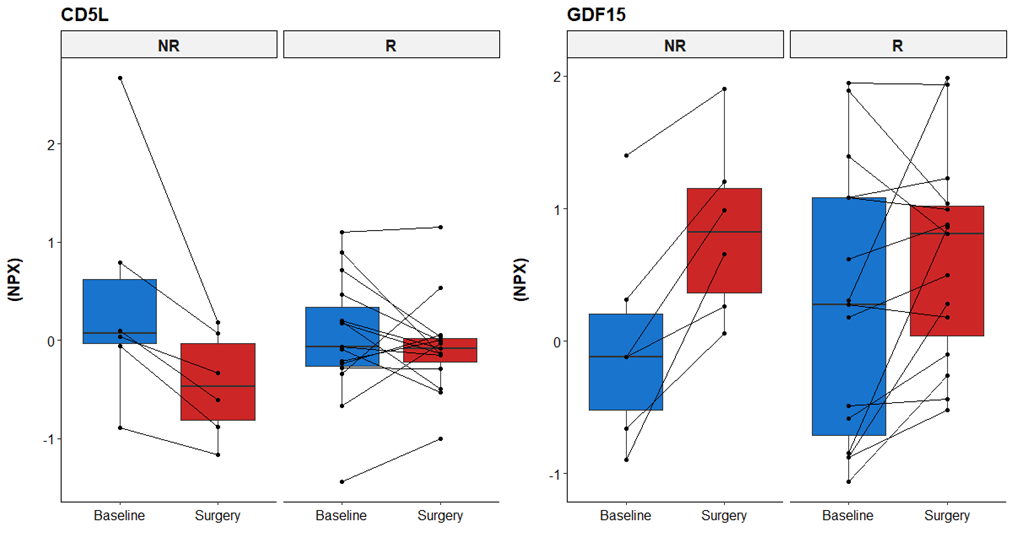
**

**Supplementary Figure 3: Proteomic profiling of plasma levels of CD5L and GDF15 proteins in UPS patients after neoadjuvant chemotherapy**
